# Can performance-based financing improve efficiency of health centers in Ethiopia? A Malmquist Productivity Index analysis

**DOI:** 10.1186/s12913-024-11127-4

**Published:** 2024-06-01

**Authors:** Mideksa Adugna, Girmaye Dinsa, Nelisiwe Khuzwayo

**Affiliations:** 1 School of Nursing and Public Health, Discipline of Public Health, University of KwaZulu-Natal, Durban, South Africa; 2 Oromia Health Bureau, Addis Ababa, Ethiopia; 3 Department of Public Health and Health Policy, College of Health Sciences, Haramaya University, Harar, Ethiopia; 4 Fenot Associates, Department of Global Health and Population, Harvard T. H. Chan School of Public Health, Addis Ababa, Ethiopia; 5 Department of Health Policy and Management, Jimma University, Jimma, Ethiopia

**Keywords:** Data envelopment analysis, Efficiency, Malmquist Productivity Index, Inputs, Outputs

## Abstract

**Introduction:**

The Ethiopian government has introduced several healthcare financing reforms intending to improve efficiency. Piloting implementation of performance-based financing is one of these actions. The purpose of this research is to assess the efficiency of healthcare facilities that have implemented performance-based financing compared to those that have not.

**Methods:**

Efficiency was measured using a nonparametric data envelopment analysis and the Malmquist Productivity Index technique. Total factor productivity change, technical change, and technological change are compared across eight sampled healthcare facilities that are implementing performance-based financing and eight that are not in Ethiopia.

**Results:**

Health facilities implementing performance-based financing have a mean technical efficiency score of 64%, allowing for a potential 36% reduction in inputs without affecting outputs. Their scale efficiency is 88%, indicating a potential 12% increase in total outputs without expanding facilities. In contrast, facilities not implementing performance-based financing have a mean technical efficiency score of 62%, with a potential for 38% input reduction without affecting outputs. Their scale efficiency is 87%, suggesting a potential 13% increase in total outputs without scaling up facilities. Among the 16 healthcare facilities observed, seven experienced a decline in the mean total productivity, while one remained stagnant. The remaining eight facilities witnessed an increase in productivity. The healthcare facilities implementing performance-based financing showed a 1.3% decrease in mean total productivity during the observed period. Among them, five showed an increase and three showed a decrease in the total factor of productivity. The mean total factor of productivity of all healthcare facilities not implementing performance-based financing remained stagnant over the three-year period (2019–2021), with four showing an increase and four showing a decrease in total productivity.

**Conclusions:**

The study concludes that implementing performance-based financing did not improve productivity levels among healthcare facilities over three years. In fact, productivity decreased among the facilities implementing performance-based financing, while those not implementing it remained stagnant. This shows health facilities that implement performance-based financing tend to utilize more resources for similar outputs, contradicting the anticipated efficiency improvement.

## Introduction

Improving efficiency is a major focus of policy makers and practitioners these days which could be achieved through minimizing waste, reducing unnecessary costs, and maximizing output given a fixed set of inputs. This has significant potential to increase the fiscal space for health. However, improvement efficiency will only be realised with a substantial effort from various stakeholders since it depends heavily on the ability of the health care facilities to implement efficiency improving policy directions. By emphasizing the way resources are allocated and utilised, the system would bring a higher health outcome with the same amount of spending or achieve the targeted outcome with a smaller amount of spending [1–3], as 20–40% of resources allocated to health worldwide is wasted for reasons related to inefficiency [1].

Efficiency seeks to improve how well health resources are utilized to achieve a specific output or outcome. This could be measured by the ratio of output to input and how effectively resources are transformed into desired results. It also focuses on minimizing waste, reducing unnecessary costs, and maximizing output given a fixed set of inputs [4].

Achieving universal health coverage requires increased resources for health, efficient use of existing resources, and more significant equity in financing and accessing quality health care [5]. Efficient resource allocation and use are two of the main focus areas of the Ethiopian Health Care Financing Strategy. Improving efficiency continues to be emphasised in Ethiopia’s Health Sector Transformation Plan II, and delivering health care that maximises resource use and avoiding waste is one of the dimensions of quality highlighted in this plan [6, 7]. In Ethiopia, healthcare financing reforms has introduced to improve efficiency. One of their major agendas is the implementation of performance-based financing (PBF).

PBF is a form of payment characterized by incentives provided to health service providers. Payments depend explicitly on the efforts of providers to achieve specific pre-established targets. It helps to streamline the focus of health system objectives when purchasers, funders, or the government can choose payment indicators that should be health priorities [8–10]. This financing approach mainly addresses health service access and coverage gaps through a health system-strengthening approach. However, there needs to be more empirical evidence regarding the efficiency of health centers under this approach in delivering health services [11, 12]. A health facility is thought to be efficient if it produces the maximum output using existing resource or maintain the current production level with a reduced magnitude of inputs [13]. Little research has been conducted to determine whether the PBF approach improved productivity in healthcare facilities. Therefore, the study seeks to contribute to the existing research gaps in the area. This research evaluates the change in efficiency of health facilities implementing PBF and those not implementing PBF for three years.

## Methods

### Study design and setting

An institution-based cross-sectional study design was employed. The focus was on 16 health centers, of which eight were implementing PBF, and the remaining eight were not implementing PBF. All the health centers selected were from the Borena zone in the Oromia region. Health facilities are chosen from districts that share similarities in terms of socio-economic status, livelihoods of the community in the catchment area, and geographic characteristics. The source of data for this study was records from the health facilities, and expenditure data was collected from the District Finance Offices. Trained data collectors collected the data.

### Conceptual framework

In this study we used a total factor of productivity (TFP) to measure the overall efficiency with which multiple inputs are transformed into outputs. The Malmquist Productivity Index (MPI) is used to assess changes in productivity or measure the overall output/results achieved about the input or resources utilized over time. It compares the productivity levels of two different periods by considering both efficiency change and technological change. Technological change refers to advancements and improvements in technology, techniques, or production methods that increase productivity and efficiency. Technical change refers to changes in the level of efficiency in production processes. It measures improvements or deterioration in converting inputs into outputs without considering technological changes. Scale efficiency refers to the optimal utilization of resources at a given scale of operation. Pure efficiency refers to the ability of healthcare facilities to produce the maximum output from a given set of inputs.

Data Envelopment Analysis (DEA), a mathematical programming-based method, was used to assess the efficiency of health centers in producing the MPI. This method converts multiple input and output measures into a single summary measure. Technically, the MPI was developed to measure changes in productivity over time and can be analysed from a technical change and technological change point of view, as suggested by Grosskopf, Norris, and Zhang (1994) [14]. The TFP decomposition into technical change and efficiency change aids in tracing from where a given change in productivity emanated.

The MPI distance function of output-oriented between period t and t + 1 can be computed using the following formula:

$${\text{MPI}}_{1}^{\text{t}}=\frac{{\text{E}}_{1}^{\text{t}}\left({\text{X}}^{\text{t}+1}, {\text{Y}}^{\text{t}+1}\right)}{{\text{E}}_{1}^{\text{t}}\left({\text{X}}^{\text{t}}{\text{Y}}^{\text{t}}\right)}$$

$$\text{M}\text{P}{\text{I}}_{1}^{\text{t}+1}=\frac{{\text{E}}_{1}^{\text{t}+1}\left({\text{X}}^{\text{t}+1}, {\text{Y}}^{\text{t}+1}\right)}{{\text{E}}_{1}^{\text{t}+1}\left({\text{X}}^{\text{t}}{\text{Y}}^{\text{t}}\right)}$$

where MPI_1_^t^ and MPI_1_^t + 1^ indicate the MPI of TFP in period t and period t + 1, respectively; E_1_^t^ (X^t+1^, Y^t+1^) indicates the output distance function, which measures technological change between period t and actual data from period t + 1; E_1_^t^ (X^t^, Y^t^) indicates the output distance function, which measures period t data about technology during the same period; E_1_^t + 1^ (X^t+1^, Y^t+1^) indicates the output distance function assessing period t + 1 data about technology during the same t + 1 period; and E_1_^t + 1^ (X^t^,Y^t^) indicates the output distance function assessing period t data about technology in the t + 1 period.

The geometric means of the two MPIs give the following equation:

$$\text{M}\text{P}{\text{I}}_{1}^{\text{G}}={\left(\text{M}\text{P}{\text{I}}_{1}^{\text{t}} \text{M}\text{P}{\text{I}}_{1}^{\text{t}+1}\right)}^{1/2}={\left(\frac{{\text{E}}_{1}^{\text{t}}\left({\text{X}}^{\dot{\text{t}}+1}, {\text{Y}}^{\text{t}+1}\right){\text{E}}_{1}^{\text{t}+1}\left({\text{X}}^{\text{t}+1}{, \text{Y}}^{\text{t}+1}\right)}{{\text{E}}_{1}^{\text{t}}\left({\text{X}}^{\text{t}}{\text{Y}}^{\text{t}}\right){\text{E}}_{1}^{\text{t}+1}\left({\text{X}}^{\text{t}}{\text{Y}}^{\text{t}}\right)}\right)}^{1/2}$$

In this study, the MPI technique was employed to asses the relative technical efficiency of each primary healthcare facility in maximising health services. Spinks and Hollingsworth highlighted that the assumption of constant and variable returns to scale DEA is a precondition for constructing the MPI [15]. Accordingly, constant return to scale (CRS) and an output-oriented model are assumed in the analysis of this study since it is a cross‐sectional study with homogeneity in economic and technical aspects of the health care system under which the health care facilities operate.

A healthcare facility is considered to have improved over subsequent years when its MPI score exceeds 1, indicating increased efficiency and productivity. This signifies that the facility’s frontier productivity has shifted outward, potentially due to internal technical progress or external influences within the healthcare system.

On the other hand, a healthcare facility may experience a decline in productivity represented by an MPI score of less than 1, indicating inefficiency or a worsening performance in delivering health services. This regression could be attributed to an increase in inputs without a corresponding increase in outputs or health outcomes.

Furthermore, a healthcare facility is deemed stagnant when its MPI score is 1, indicating a lack of progress in productivity during a given time frame. This suggests that the facility has yet to experience significant improvements or declines and has maintained a relatively constant level of productivity.

### Data analysis

A Malmquist Total Factor Productivity Index was estimated using a DEA computer program, Data Envelopment Analysis Programme (DEAP) version 2.1. Bootstrapping was performed using SPSS statistical software to assess the robustness of the MPIs. Further descriptive analysis was also performed using Microsoft Excel.

## Results

Table 1 shows summary statistics of the outputs (Deliveries conducted, Ante Natal Care (ANC) Conducted, Fully Immunised children and Outpatient Department (OPD) visits) and inputs (Clinical Staff, Non-clinical Staff and Budget Utilised) variables for the three years covered in the study. There is a considerable gap between the minimum and maximum values of the output and input variables. In particular, there is a wide gap between the minimum and maximum values for output variables of the healthcare facilities that are not implementing PBF. Additionally, regarding budget utilization, the lowest utilization is for the healthcare facilities that are not implementing PBF and vice versa.

**Table 1:** Summary statistics of input and output variables, 2019–2021

	Variables	Mean	Standard deviation	Minimum	Maximum
**PBF**	Deliveries conducted	214	125	44	515
	ANC 4 conducted	255	227	33	994
	Fully immunised children	325	548	12	1,870
	OPD visits	7,274	4,185	2,384	18,911
	Clinical staff	11	4	6	20
	Non-clinical staff	7	3	4	12
	Budget utilised	2,964,760	1,301,539	1,398,420	6,505,235
**Non-PBF**	Deliveries conducted	338	212	57	847
	ANC 4 conducted	265	207	43	891
	Fully immunised children	197	278	19	1,029
	OPD visits	7,996	6,092	557	22,810
	Clinical staff	13	8	3	26
	Non-clinical staff	12	7	4	26
	Budget utilised	2,825,156	1,817,168	2,141,428	3,437,658

### Technical efficiency scores

Technical efficiency scores were produced using output distance functions evaluating each year’s output–input data for each group of health care facilities.

The overall mean CRS technical efficiency score indicates that three of the 16 healthcare facilities in the study were technically efficient, with an average score of 63%. This indicates that they could have reduced their utilization of inputs by about 37% without reducing outputs. Tables 2, 3 and 4 show the mean output-based DEA technical efficiency scores during 2019–2021 for health facilities implementing PBF, not implementing PBF and all surveyed facilities, respectively.

Additionally, three of the 16 healthcare facilities were scale-efficient, with an average scale-efficiency score of 88%. The inefficient healthcare facilities had an average score of 85%, implying that there is potential for increasing total outputs by 15% with the existing capacity.

Regarding the groups of health care facilities, the mean CRS technical efficiency score of the health care facilities implementing PBF is 64%, which implies that, on average, the healthcare facilities implementing PBF could reduce their inputs by 36% without reducing outputs. Additionally, the scale efficiency is 88%, indicating the potential to increase total outputs by 12% within the existing capacity and size. Conversely, the mean CRS technical efficiency score of the health care facilities not implementing PBF is 62%, which implies that, on average, the healthcare facilities not implementing PBF could reduce their inputs by 38% without reducing outputs. Additionally, the scale efficiency is 87%, indicating the potential to increase total outputs by 13% within the existing capacity and size.

**Table 2:** Efficiency summary – facilities implementing PBF

Firm	crste	vrste	Scale	Return to scale
1	0.62	0.758	0.819	increasing
2	1	1	1	constant
3	1	1	1	constant
4	0.825	0.844	0.978	decreasing
5	0.338	0.351	0.962	decreasing
6	0.633	1	0.633	increasing
7	0.403	0.59	0.684	increasing
8	0.339	0.353	0.962	increasing
Mean	64%	74%	88%	

*Note* crste = technical efficiency from constant return to scale DEA

vrste = technical efficiency from variable return to scale DEA

scale = scale efficiency = crste/vrste

**Table 3:** Efficiency summary – facilities not implementing PBF

Firm	crste	vrste	Scale	Return to scale
1	0.152	0.162	0.936	decreasing
2	0.948	1	0.948	decreasing
3	0.193	0.364	0.53	increasing
4	1	1	1	constant
5	0.613	0.625	0.982	increasing
6	0.755	1	0.755	decreasing
7	0.901	0.968	0.93	decreasing
8	0.412	0.467	0.883	decreasing
Mean	62%	70%	87%	

**Table 4:** Total efficiency summary

Firm	crste	vrste	Scale	Return to scale
1	0.62	0.758	0.819	increasing
2	1	1	1	constant
3	1	1	1	constant
4	0.825	0.844	0.978	decreasing
5	0.338	0.351	0.962	decreasing
6	0.633	1	0.633	increasing
7	0.403	0.59	0.684	increasing
8	0.339	0.353	0.962	increasing
9	0.152	0.162	0.936	decreasing
10	0.948	1	0.948	decreasing
11	0.193	0.364	0.53	increasing
12	1	1	1	constant
13	0.613	0.625	0.982	increasing
14	0.755	1	0.755	decreasing
15	0.901	0.968	0.93	decreasing
16	0.412	0.467	0.883	decreasing
Mean	63%	72%	88%	

### Malmquist TFP Change

The results from the decomposition of the Malmquist TFP (MTFP) index summary of the healthcare facilities are presented in Table 5. The bootstrap estimates of the means are robust and statistically significant (*p* < 0.05). The estimates are reported in technical efficiency change (EFFCH), which is divided into pure efficiency change (PECH) and scale efficiency change (SECH), respectively, and technical or technological change (TECHCH). When analysing the temporal change in productivity of healthcare facilities between 2019 and 2021 using the MTFP index, eight of the 16 healthcare facilities experienced an increase in the mean TFP. The remaining seven health care facilities witnessed a decline and one stagnant. The increase in TFP ranges from 5 to 40%, and the decline is between 1% and 41%.

A regress in technology could cause a decline in TFP during the three years. In all healthcare facilities, the technical change component of the MTFP index is less than 1 (range: 0.784 to 0.947). The healthcare facilities that registered positive efficiency changes are those implementing PBF. Additionally, the change in efficiency was mainly driven by the change in scale efficiency. Overall, TFP declined by 0.7%. This was due to a decline of 4.7% in technical change, although there was a 4.2% increase in efficiency.

**Table 5:** Malmquist Index summary of health care facilities’ means, 2019–2021

Health care facility	EFFCH	TECHCH	PECH	SECH	TFPCH
1	0.982	0.867	0.891	1.102	0.851
2	1	1.099	1	1	1.099
3	1	1.05	1	1	1.05
4	1.026	1.156	1.015	1.011	1.185
5	1.18	1.167	1.089	1.084	1.377
6	0.898	0.916	1	0.898	0.823
7	1.407	0.818	1.278	1.101	1.151
8	0.721	0.821	0.714	1.009	0.592
9	0.817	1.097	0.871	0.938	0.897
10	0.808	0.964	0.943	0.857	0.779
11	1.566	0.798	1.196	1.31	1.25
12	1	0.784	1	1	0.784
13	1.252	1.117	1	1.252	1.398
14	0.992	0.954	1	0.992	0.947
15	1.054	0.952	1.016	1.037	1.004
16	1.311	0.835	1.332	0.984	1.095
Mean	1.042	0.953	1.011	1.03	0.993

The group of healthcare facilities implementing PBF experienced a 1.3% decline in TFP with a TFPCH of 0.987 during the three years. This may be because of the regress in technology and pure efficiency change. In all the healthcare facilities, the technical change component of the MTFP index is less than one (range: 0.592 to 0.851), as indicated in Table 6. Furthermore, it is evident that the primary driver of the efficiency change was the alteration in scale efficiency.

The group of healthcare facilities not implementing PBF experienced stagnation in TFP with a TFPCH of 1 (one) during the three-year period, which may be because of the regress in technology. In this group of healthcare facilities, the technical change component of the MTFP index is less than one (range: 0.784 to 0.964), as indicated in Table 7. Furthermore, it is evident that the change in both scale efficiency and pure efficiency were the primary drivers of the overall efficiency change.

**Table 6:** Malmquist Index summary of healthcare facilities implementing PBF, 2019–2021

Health care facility	EFFCH	TECHCH	PECH	SECH	TFPCH
1	0.982	0.867	0.891	1.102	0.851
2	1	1.099	1	1	1.099
3	1	1.05	1	1	1.05
4	1.026	1.156	1.015	1.011	1.185
5	1.18	1.167	1.089	1.084	1.377
6	0.898	0.916	1	0.898	0.823
7	1.407	0.818	1.278	1.101	1.151
8	0.721	0.821	0.714	1.009	0.592
Mean	1.010	0.977	0.987	1.024	0.987

**Table 7:** Malmquist Index summary of healthcare facilities not implementing PBF, 2019–2021

Health care facility	EFFCH	TECHCH	PECH	SECH	TFPCH
1	0.817	1.097	0.871	0.938	0.897
2	0.808	0.964	0.943	0.857	0.779
3	1.566	0.798	1.196	1.31	1.25
4	1	0.784	1	1	0.784
5	1.252	1.117	1	1.252	1.398
6	0.992	0.954	1	0.992	0.947
7	1.054	0.952	1.016	1.037	1.004
8	1.311	0.835	1.332	0.984	1.095
Mean	1.074	0.930	1.036	1.037	1.000

The yearly TFP indices for each healthcare facility indicate that the second year showed a decline in TFP while the third year experienced an increase in TFP. The yearly TFP indices for each group (healthcare facilities implementing PBF and healthcare facilities not implementing PBF) indicate that the second year witnessed a decline in TFP for both groups by 3.4% and 13%, respectively. In the third year, both groups experienced an increase in TFP, with the group of healthcare facilities implementing PBF experiencing a 1% increase and those not implementing PBF experiencing a 15% increase, as indicated in the table below (Table 8).

**Table 8:** Change in Malmquist Index summary of Healthcare facilities 2019–2021

Type	Firm	Change from year 1 to 2					Change from year 2 to 3				
		EFFCH	TECHCH	PECH	SECH	TFPCH	EFFCH	TECHCH	PECH	SECH	TFPCH
PBF	1	1.3	0.7	1.1	1.2	0.9	0.7	1.1	0.7	1.0	0.8
	2	1.0	0.9	1.0	1.0	0.9	1.0	1.3	1.0	1.0	1.3
	3	1.0	0.9	1.0	1.0	0.9	1.0	1.2	1.0	1.0	1.2
	4	1.1	1.1	1.0	1.0	1.1	1.0	1.2	1.0	1.0	1.2
	5	1.5	0.9	1.2	1.2	1.3	0.9	1.5	1.0	1.0	1.4
	6	1.1	0.8	1.0	1.1	0.8	0.8	1.1	1.0	0.8	0.8
	7	2.0	0.7	1.6	1.2	1.3	1.0	1.0	1.0	1.0	1.0
	8	0.8	0.7	1.0	0.9	0.6	0.6	1.0	0.5	1.2	0.6
Mean		1.18	0.82	1.11	1.07	0.97	0.86	1.17	0.88	0.98	1.01
Non-PBF	1	0.9	1.1	1.0	0.9	1.0	0.8	1.1	0.8	1.0	0.8
	2	1.0	0.6	1.0	1.0	0.6	0.7	1.5	0.9	0.8	1.0
	3	1.2	0.6	0.5	2.3	0.7	2.1	1.0	2.8	0.8	2.1
	4	1.0	0.6	1.0	1.0	0.6	1.0	1.0	1.0	1.0	1.0
	5	1.5	0.8	1.0	1.5	1.2	1.1	1.5	1.0	1.1	1.6
	6	0.9	0.9	1.0	0.9	0.8	1.1	1.0	1.0	1.1	1.1
	7	1.1	0.9	1.0	1.1	0.9	1.0	1.1	1.0	1.0	1.1
	8	1.7	0.7	1.8	1.0	1.2	1.0	1.0	1.0	1.0	1.0
Mean		1.11	0.78	0.99	1.13	0.87	1.04	1.11	1.09	0.95	1.15

## Discussion

The study findings reveal that health facilities implementing PBF have a mean technical efficiency score of 64%, while those not implementing PBF have a score of 62% within the three years. Out of the eight healthcare facilities implementing PBF we examined, 6(75%) were found to be more technically efficient. The level of inefficiency is much lower than the findings from another zone in Ethiopia, i.e. 50% [15]. In contrast, studies in other African countries found the inefficiency worse in the studied healthcare facilities. For instance, 73% of the health centres in southwestern Uganda [16], 59% of peripheral health units in the Kailahun and Kenema districts of Sierra Leone [17], 59% of health care facilities in the Pujehun district of Sierra Leone [18] and 54.5% of public health facilities in Meru county of Kenya [19] were found to be technically inefficient. Our finding revealed better productivity compared to the inefficiency of public health centres in Ghana, i.e. 78% [20].

The level of inefficiency of the health centers in this study is almost similar to the inefficiencies in hospitals. Studies investigating the (in)efficiency of hospitals found it to be 67% [21] in Zambia and 76.2%, 76.2% and 61.9% of the studied hospitals run inefficiently in 2006, 2007 and 2008, respectively, in Botswana. In contrast, the inefficiency is worse in the studied hospitals in three provinces of South Africa, 87% [21]. Conversely, the efficiency level ranged from 26% to 37% in the studied hospitals in Namibia [22].

With another measurement aspect, the average TFP of the health centers under the study and implementing PBF is 0.987. This finding is comparable to findings obtained from hospitals studied in eastern Ethiopia, with 0.946 [26], and other countries like Benin, with 0.951 [28] and South Africa, with 0.879 [23]. Also, health facilities regress in productivity, as evidenced from Nepal with 0.93 [27], China with 0.926 [24], and Taiwan with 0.788 [25]. Conversely, with increased productivity, the TFP of health centres in the Seychelles was 1.024 [26]. Also, other studies indicate that hospitals had an average MTFP score greater than 1, signifying productivity growth, with 1.014 in Vietnam [27], 1.235 in district hospitals in India [28], and 1.121 in coastal hospitals in China [29].

The productivity results of the health centers revealed that those implementing PBF showed a decrease in productivity growth over time. Specifically, there was a 1.3% decline in productivity, which may be attributed to a 2.3% decrease in technological change and a 1.3% decrease in pure efficiency change. The results for the health centers not implementing PBF indicated uniformity (no change) in productivity growth (1.000). Therefore, the findings from this study suggest that productivity did not improve among healthcare facilities implementing PBF over the study period. This could be because healthcare facilities implementing PBF may allocate excessive inputs or resources to achieve the intended targets [30]. However, further studies should investigate aspects other than input utilization [31, 32].

## Conclusion

This study provides evidence on productivity change that contains efficiency and technical change using data from 16 health centers. The health centers studied belong to the group that implementing PBF and not implementing PBF from 2019 to 2021. This study found that the level of TFP of the health care facilities implementing PBF decreased over time. In contrast, the health facilities that did not implement PBF witnessed stagnation in TFP. Based on the analysis, PBF hasn’t improved the productivity of healthcare facilities over the years.

### Limitations of the study

- The analysis reported in this paper depends on data from 2019 to 2021. Therefore, the status of health care facilities may have changed significantly.
- Spending for health service providers other than the regular budget i.e. resources received in the form of grants and support is not included in the study.

## Data Availability

The datasets used and analysis for the study are available from the corresponding author upon reasonable request.

## Abbreviations

ANC: Ante natal care
CRS: Constant return to scale
DEA: Data envelopment analysis
DEAP: Data envelopment analysis programme
EFFCH: Technical efficiency change
MPI: Malmquist productivity index
OPD: Outpatient department
PBF: Performance-based financing
PECH: Pure efficiency change
SECH: Scale efficiency change
TECHCH: Technological change
TFP: Total factor of productivity
TFPCH: Total factor of productivity change
VRTe: Technical efficiency from variable return to scale
